# Moral enhancement and the good life

**DOI:** 10.1007/s11019-018-9868-4

**Published:** 2018-09-24

**Authors:** Hazem Zohny

**Affiliations:** The Oxford Uehiro Centre for Practical Ethics, The University of Oxford, Suite 8, Littlegate House, 16-17 St Ebbe St, Oxford, OX11PT UK

**Keywords:** Enhancement, Well-being, Ethics

## Abstract

One approach to defining enhancement is in the form of bodily or mental changes that tend to improve a person’s well-being. Such a “welfarist account”, however, seems to conflict with moral enhancement: consider an intervention that improves someone’s moral motives but which ultimately diminishes their well-being. According to the welfarist account, this would not be an instance of enhancement—in fact, as I argue, it would count as a disability. This seems to pose a serious limitation for the account. Here, I elaborate on this limitation and argue that, despite it, there is a crucial role for such a welfarist account to play in our practical deliberations about moral enhancement. I do this by exploring four scenarios where a person’s motives are improved at the cost of their well-being. A framework emerges from these scenarios which can clarify disagreements about moral enhancement and help sharpen arguments for and against it.

One way to understand the concept of enhancement is to associate it with well-being promotion: to be enhanced is merely to change your body or mind in ways that tends to improve your well-being (Savulescu et al 2011). There is an appealing simplicity to this approach: rather than getting bogged down trying to non-arbitrarily define the boundaries of normal functioning and what exceeds it, let us instead forgo this ultimately irrelevant exercise and focus on defining enhancement by what seems more practically significant, which is how bodily and mental changes impact well-being. After all, if we are interested in being smarter, or stronger, or longer-lived, it is presumably because such changes to our capacities would be good for us in some sense—that is, good for our well-being. Indeed, if it turns out such changes aren’t good for us, then perhaps we shouldn’t call them enhancements at all (Harris 2007).

But consider moral enhancement: becoming more moral—whether through a hypothetical biomedical intervention or more traditionally via some form of education or training—can potentially diminish a person’s well-being. Perhaps this is because having the right motives or doing the right thing can often entail a reduction in autonomy or pleasure, or the frustration of one’s own interests or desires more generally. This seems plausible: moral acts can sometimes require a significant sacrifice of one’s own interests and, to that extent, of one’s well-being.

While this is a contestable claim, if it is correct, it suggests that a well-being-centred (or welfarist) approach to enhancement is limited in its conceptual scope—it can account for moral enhancement *only* when it tends to be beneficial to the interests of the person undergoing that moral enhancement. If a moral improvement leaves a person with better moral motives but simultaneously reduces how well their life goes overall, then according to a welfarist account of enhancement they have not been enhanced in any sense. In fact, as I will argue, the most fully developed welfarist account would conclude that this change in motives has led to a disability in the person—*even* though they have been improved morally.

Here, I investigate this seeming tension between offering a general account of enhancement that is rooted in individual well-being promotion and a plausible account of moral enhancement. I will argue that, despite differences between the self-regarding nature of enhancing capacities such as our physical and cognitive traits, and enhancing other-regarding capacities, such as our moral dispositions and behaviours, the individual-focused welfarist account nevertheless can play an insightful and ultimately fruitful role in thinking about moral enhancement and its implications. Specifically, it is a role that can be played alongside more traditional and intuitive approaches to moral enhancement, which I will call moral value-based accounts.

My case for this welfarist approach to moral enhancement unfolds in three parts. First, I more fully introduce the welfarist account of enhancement and disability developed by Savulescu and colleagues (2011) and elaborate on the apparent conflict between it and moral enhancement. I then argue that while we may wish to distinguish welfarist enhancement from moral enhancement, those two are likely to conflict far less often than might be assumed. To do this, I use a number of examples to demonstrate how the welfarist account has a central role to play in helping ethicists and policy makers assess different forms of interventions that might impact our moral motives and behaviours. Finally, I argue for the usefulness of the welfarist account in clarifying some of the disagreements surrounding moral enhancement, specifically those disagreements centred on whether moral enhancements might diminish the autonomy of those who make use of them.

## The welfarist account and moral enhancement

According to the welfarist account, an enhancement is any change in the biology or psychology of a person which *increases* the chances of that person leading a good life in the relevant set of circumstances (Savulescu et al. 2011). Savulescu and colleagues couple this with a welfarist account of disability, which defines a disability as any change in the biology or psychology of a person which *decreases* the chances of leading a good life in the relevant set of circumstances.

In this way, an enhanced bodily or mental state is an advantageous state for a person to be in, while a disabled one is a disadvantageous state. This means that, if taking a drug augments some function but this augmentation does not tend to benefit well-being, then this intervention is not an enhancement (Earp et al. 2014). Similarly, if a condition technically impairs a bodily or mental function but that condition nevertheless does not tend to negatively impact well-being, then it is not a disability.

The idea here is, in part, to disentangle the concepts of enhancement and disability from further controversial concepts like normality, health and disease, which themselves are the subjects of much disagreement. The welfarist approach also allows us to home in on what seems more relevant to our practical deliberations about whether and how to intervene in a person’s biology or psychology: the likely well-being of the individual who undergoes a change to their body or mind (as opposed to how that change medically relates to normal functioning or health).

To be sure, this approach has a number of counter-intuitive implications even outside the realm of moral enhancement. It has been criticized on multiple grounds, with worries about how it stretches the concepts of enhancement and disability far beyond their everyday language use, how it makes no distinction between treatment and enhancement, how it relies on the contested notion of a “good life”, how it is overly individualist, and how it seems to sidestep other ethical issues raised by these topics (Coenen et al. 2011; Sparrow 2013; Camporesi 2014; Gordon 2014; Beck and Stroop 2015). These criticisms have been responded to elsewhere (Zohny 2014, 2016) and I will not explore those responses here. Instead, I will proceed on the assumption that for non-moral enhancement, the welfarist account is at least coherent, if not plausible and attractive for the reasons mentioned above.

However, I want to first highlight what is meant by a “good life” in this context. This term can be misleading, as it can be interpreted to mean “a morally good life” or at least a life of well-being that is also moral. And while there is a dispute in theories of well-being as to whether moral virtue is good for an agent (Hooker 1996), for the welfarist account, and in line with most theories of well-being, the meaning of a good life here is limited solely to what is good *for* a person having that life. This variety of good is called prudential value, which we can contrast with moral value—things that are good from the view point of morality, as opposed to a self-regarding view point (Chang 2004).

In that way, the welfarist account is rooted in prudential value: it is about what is good *for* a person in terms of changes to their body or mind. Depending on the specific account, this goodness may be expressed in terms of pleasure, autonomy, meaningfulness, the satisfaction or fulfilment of their desires, the attainment of other goods like knowledge or friendship—but it typically does not include what may be good independently of that person’s well-being. Hence, it is not a moralized account of enhancement—it is not concerned with *moral* value.

With this in mind, we can start to see the difficulty the welfarist account might have with moral enhancement specifically. A straightforward way to define moral enhancement is as an intervention that leaves an individual with morally better motives or behaviours than they otherwise would have had (e.g. Douglas 2013). This notion of being *more* moral reflects two things. Firstly, it suggests an augmentative understanding of moral enhancement: there is a boost in our capacities to have more moral motives and and/or to behave more morally without reference to how this impacts our own well-being. Secondly, it appeals to moral rather than prudential value: the defining feature is not how it impacts the person’s well-being but how it impacts their moral motives and behaviours.

In contrast to this moral value-based definition, how might the welfarist account define moral enhancement? To the extent that our moral motives and dispositions are part of, or result from, our psychology, they would seem to be covered under the broad definition of enhancement as a well-being improving change to our psychology. More specifically, we might articulate it as: Any change to the moral motives of a person which increases their chances of leading a good life in the relevant set of circumstances. We can change moral motives with moral capacities or moral behaviours if we wish. But, whichever way we articulate it, we can see the implausibility of the above characterization of moral enhancement.

This implausibility stems from the fact that, according to such a definition, for something to count as a moral enhancement, it must tend to be advantageous to the person who has undergone the alteration. In fact, if one becomes more moral and yet this *decreases* their chances of leading a good life, they have become disabled. Such a moral change is disadvantageous for them, even if it makes them morally better.

This seems a strange way of articulating the nature and value of moral enhancement; it arguably limits the scope or generality of the welfarist account. It suggests the account simply misclassifies interventions and lacks the conceptual resources to intuitively or successfully refer to changes to an individual’s dispositions or behaviours that make them morally better, but that leave them expectably worse off. Indeed, some of the architects of the welfarist account seem to conclude as much, quietly disregarding it in favour of a moral-valued-based approach to moral enhancement, where improving or augmenting our moral capacities is what constitutes moral enhancement (Earp et al. 2017; Persson and Savulescu 2008, 2012).

Nevertheless, we may plausibly wish to resist the notion that prudential value conflicts with moral value in this way. Many traits associated with being moral can indeed make our lives go better: leading a more tolerant, patient, honest and understanding life would seem to be a life with less frustration and hatred in it, and possibly more friendship and solidarity, and in that sense improving these traits associated with moral dispositions in a person who lacks them could be advantageous for them prudentially speaking.

This may be true, and to the extent that it is, there is no conflict between welfarist enhancement and moral enhancement. Yet it is not clear that other traits associated with being moral, such as being compassionate or selfless, are necessarily advantageous in that prudential sense. These may entail leading a more difficult or burdensome life. We can see this in the fact that most of us would likely at least hesitate to undergo some perfectly safe intervention that might mean we will more intensely wish to, say, live more frugally, or donate much of our wealth, or go on vacation less often, or spend more time volunteering in night shelters. We would hesitate precisely because it’s not clear that such a change to our lives would be advantageous from a prudential perspective. And so, if we were to nevertheless undergo such an intervention, it is likely to be motivated by something other than the promotion of our own well-being. In that way, moral enhancement does seem fundamentally different from the potentially welfarist enhancements associated with, say, longevity or increased strength.

Because of this, we may wish to distinguish between welfarist enhancement (which is best understood as self-regarding), and moral enhancement (which is not necessarily self-regarding) as two separate domains of enhancement. Here, welfarist enhancement may encompass all forms of self-regarding enhancements to oneself (be they physical, aesthetic, genetic, cognitive, mood-based), but not moral enhancements. Moral enhancement is different in the sense that it can be best understood as augmenting moral value, as it typically is in the associated literature.

If this is correct, then one immediate conclusion is that the welfarist account is not in fact a general account of enhancement: its scope is limited in a way that its original formulators have not explicitly recognized.

However, to conclude from this that the welfarist account is simply not relevant to the discussion on moral enhancement would be mistaken. In the next section, I argue that, despite its limitations in making common sense classifications about moral enhancements, it can nevertheless play a crucial role in our *practical* analyses of them. In fact, as we will see, much of the seeming incompatibility between welfarist and moral enhancement boils down to articulating that incompatibility in abstract terms—such as in the form of “prudential versus moral value”. Once we consider concrete scenarios where we might expect to witness this conflict, a fundamental role for the welfarist account emerges.

## When moral enhancement is bad for you

Here, I present four scenarios where a person’s moral motives and behaviours are seemingly improved, but where they personally become worse off. I argue the welfarist account’s referral to the interventions in these scenarios as disabling is correct, and reflects an important dimension to our assessment of morality-altering interventions that is lacking in moral value-based accounts of moral enhancement such as Douglas’s (2013). In what follows, note that I am not speculating about the possibility or likelihood of these hypothetical interventions being developed—instead, I refer to them merely to draw observations about the scope of the welfarist account.

The clearest example of improved moral motives or behaviours that leave a person worse off is sacrificing one’s life to save another’s. In such a case, the cost to the individual is overwhelming: not only might the action be costly in terms of the pain or fear entailed by the process of dying, but it is supremely costly in that they lose the capacity for well-being altogether.^1^ And yet, such an act would ordinarily be understood as moral, if not heroically so.

Based on this, suppose a drug is invented, call it the Jesus drug: you take it and suddenly feel highly motivated to find a way to sacrifice your life to save others. You track down terminal patients who need a heart, liver, and lungs and with whom you match. You notify the relevant authorities, and kill yourself in the appropriate place, saving three others.

The Jesus drug, as is quite clear, is prudentially highly disadvantageous for its user. True, it augments a particular, if controversial, moral motive (sacrificing yourself for others), but that is clearly not its most salient feature: its most salient feature is that it makes its users end their lives very quickly and reliably. Calling it a moral enhancer merely because it augments a certain moral motive seems to be missing the more central feature of what it does. The welfarist account, on the other hand, which would label it a disabling drug, perfectly captures this central feature. In other words, the welfarist account is able to reflect what is disturbing about such a drug in a way that a moral value-based account of moral enhancement does not.

Of course, perhaps morally good motivation requires reaching decisions more deliberately—the Jesus drug just creates one overriding desire to sacrifice one’s life for others, and in that sense we may argue it does not qualify as a moral enhancer. If so, then this is not an instance of being morally enhanced that is disadvantageous, and thus does not reflect a conflict between welfarist enhancement and moral enhancement.

Let us therefore consider a slightly less extreme case. Suppose an individual, call her Beneficent, hears about a new drug, *Altruix*, that promises to improve her moral motives and behaviours. Wanting to be a morally better person, Beneficent starts taking it and soon after notices that she wants to be kinder, more understanding and generous, and so begins behaving in those ways more and more often. These changes improve the lives of people she comes into contact with (as she is a much more caring and helpful person now), and this in turn improves her own life too: there appears to be more love in her life and she feels she contributes to others in a way that is meaningful. A sense of purpose ensues that far outweighs any of the sacrifices and efforts entailed by being more caring and giving. Up until now, her increased moral actions have also been prudentially advantageous to her. In other words, Altruix appears to be a moral enhancer and a welfarist enhancer.

However, as Beneficent continues to take *Altruix*, things get out of hand: she begins donating more and more of her time and income to others, losing touch with friends and family. Most of her furniture she sells and donates the money. Eventually, she donates a kidney to a stranger. In fact, she finds that over the coming months she keeps herself only as healthy and wealthy as is necessary to maximize her ability to help others. What’s more, these further developments are now severely diminishing her happiness: she feels constantly exhausted and in pain, crying herself to sleep every night at the thought of others’ suffering. More than anything, she now feels deeply alone in the world as few others share her concerns in the same way. Nevertheless, there is no doubt her actions continue to improve the lives of people around her (and all around the world where she has sent her donations).

Clearly, Beneficent, while she continues to create moral value in the world through her actions, has suffered a significant diminishment to her well-being.^2^ From a prudential perspective, she was better off before taking *Altruix*. With that in mind, note that the welfarist account seems very capable of capturing this fact about Beneficent’s life: the use of *Altruix* has had a disabling effect on her. It has proved to be a disadvantageous change to her psychology. Is there anything particularly lacking about this conclusion? Indeed, this seems to be the more salient fact about what *Altruix* does to its users. The welfarist account does not need to deny *Altruix* is a morality altering or improving intervention, and we may wish to call it a moral enhancer in the moral value sense—the drug does indeed augment people’s disposition to behave in more moral ways (broadly construed). Yet merely calling it a moral enhancer seems to be missing something fundamental about what the drug does: it eventually destroys the lives of people who take it. From the practical perspective of deciding to make such a drug and provide it to people like Beneficent, surely that is the salient feature worth highlighting about this drug: what people need to know about it is that, first and foremost, it will destroy their lives, not improve their moral dispositions. The fact that it may be a “moral enhancer” in the moral value-based sense is relatively irrelevant. In that way, the welfarist account plays a central role in capturing what this drug really does.

Another scenario can further convey this point: Imagine Pido, a paedophile, is regularly injected with a drug against his will that makes him lose all urges to abuse children. He now has better (or at least less bad) motives towards them. However, this drug acts on a group of neurotransmitters central to mood regulation. As a side-effect, Pido finds that while he is freed from those urges and from the risks of incarceration and/or further ostracisation, he now suffers from deep depression—he does not eat or bathe, and seems to have simply lost the will to live. His improved moral motives have come at a greater prudential cost to himself.

Again, we seem to have an instance of what is arguably a moral enhancer that is disabling from a welfarist perspective. Note that a society (or even Pido himself) could decide it has good reasons to “disable” Pido in this way despite its effects on him, and the welfarist account is not incompatible with such a conclusion. That is, this may be a case where severely diminishing someone’s well-being is the right action, all things considered. But note also how the welfarist account highlights what should arguably be occupying the bulk of our ethical deliberations in such a case: should we severely diminish a person’s well-being in order to reduce the chances of him harming children in the future? A purely moral value-based approach to moral enhancement is unable to be nuanced in this way. It does nothing to bring out what is at stake exactly nor highlight the difference between changes to moral motives that ruin a life or improve it. In that way, the welfarist account, even though it limits its scope to self-regarding interventions, plays an important role here, creating the kind of framework conducive to deliberating clearly about what is at stake exactly: the chances of a person leading a good life and its relation to broader values (be it justice, reducing overall harm, and so on).

Perhaps, however, these are all easy cases. What about a moral improvement to a person that only leaves them very slightly worse off? Is the loss in prudential value in this case still the salient feature of such an intervention? The first thing to note here is that it is not easy to articulate what such a case entails. Suppose Grumpy is an elderly man who is a little racist and does not like to share his food or help his neighbours. He’s given a course of *Altruix Light* and becomes ever so slightly more tolerable: he starts waving rather than frowning at the Filipino couple next door and his wife actually enjoys his company now. He himself starts to appreciate his wife and local community more. Despite this, we can imagine he has become slightly worse off than before: perhaps as a side effect he now spends less time alone and therefore works on his carpentry less often, and so ends up making less beautiful things in his life. Without him realising it, the drug has also been the cause of regular headaches that now diminish his ability to enjoy reading and learning new things.

The problem with cases like Grumpy’s is that they force us to adopt a very clear conception of well-being in order to evaluate them. In the previous cases, some overlapping consensus about what well-being entails was sufficient to conclude the interventions in them were clearly well-being diminishing for the individuals involved. Here, it seems like we might get different answers about how well Grumpy’s life is going now depending on whether we are hedonists, desire satisfactionists, or list theorists (and for the list theorist, the contents of that list and how we weigh up its constituents is crucial).

Nevertheless, the fact that the welfarist account requires us to become clearer about what well-being is in such cases (and how we ought to weigh it against promoting other values) seems to me a valuable feature of the account that is relevant to moral enhancement. While some individuals may laudably wish to trade some welfare for being more moral, surely whether *Altruix Light* should be made available ought to depend on how it impacts the well-being of those who use it. If it will reliably make users’ lives go worse while only marginally improving moral motives and behaviours, it may be hard to justify its promotion or use. And the reason for that is precisely because of its impact on prudential value. And so, again, the welfarist account encourages us to keep the evaluative focus on the more relevant issue here. Moral value-based approaches to moral enhancement, in and of themselves, are not capable of this.

Of course, an alternative here is, after some further thought, we conclude that, overall, the negative and positive effects of *Altruix Light* on Grumpy’s well-being cancel each other out: it has no effect on his well-being overall. In such a case, the welfarist account would not label *Altruix Light* as enhancing or disabling. It would be a morality-altering intervention that simply has no noteworthy effect on the user’s well-being. Here, it is precisely such cases that would highlight the welfarist account’s lack of conceptual resources to capture everything there is to say about moral enhancements: when a moral enhancer has no implications for a user’s well-being, the account is silent. This gives us further reason to distinguish welfarist enhancement from moral enhancement, ultimately viewing them as complementary approaches to assessing a given intervention.

Still, it is worth emphasizing how difficult it is to imagine an intervention that alters a person’s motives or behaviours without either some net benefit or cost to them. Perhaps this will be the case for individuals in very specific circumstances with very specific interests or values, but it seems implausible to conceive of a morality-altering intervention used by, say, millions which consistently has a prudentially-neutral effect on their lives. Much more plausible is that it will either be disabling or enhancing for most users.

## Clarifying disagreements

In the four scenarios above we saw how, despite the welfarist account’s inability to seemingly successfully refer to moral enhancement in general, it nevertheless helps foster a framework for thinking more clearly about morality-altering interventions. To the extent that a moral enhancer might be good for its recipient, it falls within the scope of the welfarist account; to the extent that a moral enhancer might be bad for its recipient, then the welfarist account directs us to what seems the most pertinent question: when is it justified to diminish a person’s well-being for the benefit of others?

Here I suggest this framework can play a useful role in the debate on moral enhancement, specifically by helping to clarify some of the disagreements in the associated literature. For instance, an ongoing dispute about moral enhancement is whether interventions that directly act on people’s attitudes and motivations are permissible (Agar 2014; DeGrazia 2014; Douglas 2014; Harris 2011, 2014, 2016; Persson and Savulescu 2016a, b). Harris (2011) argues interventions that would tinker with people’s attitudes and emotions so as to increase the probability they will act in ways deemed ethical would rob them of important aspects of their autonomy—namely, their “freedom to fall”, which I take to mean the ability to choose to do wrong.

Note the nature of the concern expressed here: although it is not explicitly stated, it is clearly a concern about the well-being of those who might lose the freedom to fall thanks to such interventions. That is, the force of this objection rests on the presumption that a loss of autonomy entails a loss of an ability to lead a prudentially good life. If this is the correct way to interpret Harris, then it seems helpful to understand this argument as an ultimately welfarist one—that is, one which is concerned with the well-being of the users or recipients of such moral enhancers.

To take another example: Schaefer (2015) raises a variation of this concern where moral enhancement may lead to the loss of individuality, which he defines in Millian terms as our capacity to hold beliefs and motives that are our own—that is, ones we have cultivated or developed ourselves rather than merely conformed to. Lacking such individuality could reduce us or bring us closer to the state of passive, mechanistic beings vulnerable to the instrumental control of others. In that way, if people’s moral dispositions are directly modulated through some intervention, this may reduce us to just that: mechanistic beings incapable of moral disagreement. Again, this concern, to the extent that it is valid, seems best understood as ultimately a concern about the potential threat moral enhancement may pose to our autonomy, and ultimately our capacity to lead prudentially good lives.

While I do not intend to weigh in on whether such interventions would be prudentially costly in those ways (that is, that they might in fact rob people of their autonomy or individuality), it seems useful for ethicists and policy maker to be able to clarify those concerns in welfarist terms: if Harris and Schaefer are correct, then such interventions may be severely disabling to users, even if they do improve moral dispositions or behaviours.

In response, their critics may argue that reducing the chances of great harm in the world may be worth the prudential cost of slightly diminishing users’ autonomy or individuality (Persson and Savulescu 2016a, 2017), while others may argue such interventions would not in fact reduce autonomy in any relevant way since we are not the authors of our moral dispositions anyway, and therefore moral enhancement of this kind would not be an added prudential cost (DeGrazia 2014).

Setting out the dispute in these terms helps to clarify what these disagreements are about, which is how moral enhancers are likely to impact the well-being of users.^3^ Immediately what exactly is at stake is much clearer. And here the language of the welfarist account is key. We should be able to distinguish advantageous (or well-being promoting) moral enhancers, disadvantageous moral enhancers, and perhaps prudentially-neutral ones, too.

In fact, it is worth spelling out some of the possible relationships between prudential and moral value when it comes to bodily or mental changes in a person. As you can see from Table 1, those relationships can be articulated in a least six ways, with a subset for each of those. Hence, consider a change to a person’s biology or psychology that leaves them prudentially advantaged while also increasing moral value. An example of this is vaccines, which can be advantageous not only for you, but for others as well, who are less likely to get sick precisely because you are less likely to get sick. But we can also distinguish a subset of such changes (1A) that are prudentially advantageous and also increase moral value, but which do so in a specific way *through the behaviour* of the affected individual. We can term these interventions welfarist moral enhancements. There may be many such examples, such as becoming kinder, more altruistic, more generous, and so on. These are welfarist moral enhancements only to the extent they also tend to improve the well-being of the individual compared to how they otherwise would have been.

**Table 1:** Ways biomedical interventions can impact prudential and moral value

Changes to an individual’s biology or psychology can be:	
1	Prudentially advantageous while increasing moral value (e.g. vaccines)
	• (A) Prudentially advantageous while promoting moral acts (e.g. being more altruistic)
2	Prudentially advantageous while reducing moral value
	• (A) Prudentially advantageous while impeding moral acts
3	Prudentially disadvantageous while increasing moral value
	• (A) Prudentially disadvantageous while promoting moral acts
4	Prudentially disadvantageous while reducing moral value
	• (A) Prudentially disadvantageous while impeding moral acts
5	Prudentially neutral while increasing moral value
	• (A) Prudentially neutral while promoting moral acts
6	Prudentially neutral while decreasing moral value
	• (A) Prudentially neutral while impeding moral acts

We can start to see a framework emerge from these divisions that can help us identify more clearly the nature of a concern or objection about moral enhancement. For instance, Harris (2011) and Schaefer (2015) could be understood as primarily worried that some moral enhancers might be extreme instances of 3A: they may make us merely behave more morally in a purely consequentialist sense, but with an overwhelming cost to users’ well-being (i.e. by robbing them of autonomy). Others may disagree about their prudential impact being that extreme, or might even agree but conclude that it is nevertheless a worthwhile cost. Similarly, there may be strong disagreement about 2: prudentially advantageous changes that reduce moral value. These are controversial welfarist enhancements, such as changes to the body or mind that confer a purely competitive advantage but that ultimately lead to more discrimination in a society (e.g. cosmetic changes that alter one’s racial features or sexual orientation in a way that may be beneficial for them, but that exacerbates pre-existing prejudice in a society). As for those arguing moral enhancement is doomed to backfire for both users and for creating moral value, they refer to 4: prudentially disadvantageous interventions that also decrease moral value.

An ideal starts to emerge from this that perhaps all parties can agree to: moral enhancers that are also welfarist enhancers (1A). These would be instances of “win–win” morality-altering interventions, where the two wins refer to prudential and moral value. This is indeed how we tend to think of moral improvements in our lives: they are good from a moral perspective but they are also good for us in a self-regarding sense. Whether such an end can be achieved through biomedical means is another matter, but here at least is a way of framing the possibilities of, and concerns about, moral enhancement that seems fruitful to clear deliberation.

## Conclusion

I initially argued we may wish to distinguish welfarist enhancement from moral enhancement: the former appears to lack the conceptual resources to intuitively refer to changes to an individual’s motives or behaviours that make them morally better, but that leave them expectably worse off. Nevertheless, the scenarios envisioned in the four cases of the Jesus drug, Beneficent, Pido and Grumpy, demonstrate how, despite this limitation, the account still has a crucial role to play in our practical assessments of morality-altering interventions. Those scenarios all dealt with interventions that fall under disabling but morally improving changes to a person. It is true, the welfarist account provides no *prima facie* reason to choose these; in fact, it gives us a reason to be suspicious of them. But, as I have argued, that is surely a strength of the account: any intervention that will expectably make someone worse off *should* give us pause. Moreover, if the labels we use ought to capture what is most salient about something, the welfarist account does so by labelling such interventions as disabling or disadvantageous for the user. This is not to deny there might be cases where it is unclear what is more salient, as in the case of Grumpy. In such a case, the welfarist account at least guides our attention to what seems most relevant in deciding whether to make or distribute such an intervention: the impact on the individual who takes *Altruix Light*.

In line with this, I argued we can use this account to create a framework for using prudential and moral value to clarify the nature of a concern about moral enhancement. This way of formulating some of the positions on moral enhancement can go a long way in clarifying the nature of disagreements about this contentious topic.

## Footnotes

1 Unless one believes in an afterlife—however, I am referring to death here as the cessation of an individual’s consciousness, not its transference to some other alleged realm.

2 We can envision a deontological version of this scenario. Here, taking *Altruix* motivates her to live by a categorical imperative or certain set of duties in a way that similarly diminishes her personal well-being.

3 That, at least, is one dimension of the debate. Much of the discussion also revolves around a suspicion that moral enhancements would backfire—that is, they would in fact fail to actually make us more moral (Agar 2014; Harris 2011).
